# Ultralight Industrial Bamboo Residue-Derived Holocellulose Thermal Insulation Aerogels with Hydrophobic and Fire Resistant Properties

**DOI:** 10.3390/ma13020477

**Published:** 2020-01-19

**Authors:** Hanxiao Huang, Yunshui Yu, Yan Qing, Xiaofeng Zhang, Jia Cui, Hankun Wang

**Affiliations:** 1 College of Material Science and Engineering, Central South University of Forestry and Technology, Changsha 410004, China; 2 Institute of New Bamboo and Rattan Based Biomaterials, International Center for Bamboo and Rattan, Beijing 100102, China; 3 SFA and Beijing Co-built Key Laboratory of Bamboo and Rattan Science & Technology, State Forestry and Grassland Administration, Beijing 100102, China

**Keywords:** industrial bamboo residue, holocellulose aerogel, hydrophobicity, fire resistance, thermal insulation material

## Abstract

In this study, water-soluble ammonium polyphosphate- (APP) and methyl trimethoxysilane (MTMS)-modified industrial bamboo residue (IBR)-derived holocellulose nanofibrils (HCNF/APP/MTMS) were used as the raw materials to prepare aerogels in a freeze-drying process. Synthetically modified aerogels were confirmed by Fourier transform infrared spectroscopy, X-ray diffraction, and thermal stability measurements. As-prepared HCNF/APP/MTMS aerogels showed themselves to be soft and flexible. The scanning electron microscopy (SEM) analysis showed that the foam-like structure translates into a 3D network structure from HCNF aerogels to HCNF/APP/MTMS aerogels. The compressive modules of the HCNF/APP/MTMS aerogels were decreased from 38 kPa to 8.9 kPa with a density in the range of 12.04–28.54 kg/m^3^, which was due to the structural change caused by the addition of APP and MTMS. Compared with HCNF aerogels, HCNF/APP/MTMS aerogels showed a high hydrophobicity, in which the water contact angle was 130°, and great flame retardant properties. The peak of heat release rate (pHRR) and total smoke production (TSP) decreased from 466.6 to 219.1 kW/m^2^ and 0.18 to 0.04 m^2^, respectively, meanwhile, the fire growth rate (FIGRA) decreased to 8.76 kW/s·m^2^. The thermal conductivity of the HCNF/APP/MTMS aerogels was 0.039 W/m·K. All results indicated the prepared aerogels should be expected to show great potential for thermally insulative materials.

## 1. Introduction

In recent years, significant industrial waste from cellulose has been produced annually [1]. Typically, the industrial waste is landfilled or burned, which has caused a large amount of cellulose resources to be wasted as well as environmental pollution [2,3]. As the people began to pay attention to sustainable development of the environment, some industrial waste was then recycled and used for low-cost preparation of bio-materials. Bamboo is a biomass resource with fast growth and vast availability [4,5]. It is also the second most important lignocellulosic material behind wood [6,7]. With the development of the bamboo industry, the parenchyma cells of bamboo are often discarded because of their loose structure, leading to a large amount of bamboo waste [8]. However, the loose structure of parenchyma tissue is beneficial to chemical treatment and mechanical fibrillation. 80% of industrial bamboo residue (IBR) is parenchyma tissue, which can be used to prepare cellulose nanofibers inexpensively [9]. Therefore, the application of IBR will effectively increase the added value of bamboo.

One potential method for increasing usage of IBR is to prepare cellulose aerogels, which is a solid biomass material that replaces the liquid in gels with gas without changing the 3D network structure or volume of the gel [10]. As a next generation material, cellulose aerogels overcome the fragility of silicon aerogels and can be self-assembled without a crosslinking agent. Over the past few decades, cellulose aerogels have garnered significant attention in the field of thermal insulation, because of their specific surface areas, low density, high porosity, favorable biodegradability, and biocompatibility [11]. However, the abundant hydroxyl groups and flammable properties of cellulose aerogels result in poor water and fire resistance. In thermal insulation applications, this inherent defect hinders the utilization of bio-based aerogels. But then again, abundant hydroxyl groups in cellulose aerogels provide powerful conditions for modification [12]. To improve water and fire resistance, physical or chemical modifications via reinforcing components are effective modification methods. Many methods have been reported regarding flame retardant and hydrophobic modification, including: (1) cellulose nanofibril (CNF) aerogels were modified with methylene diphenyl diisocyanate (MDI) by solvent exchange [13]; (2) CNF aerogels were crosslinked with ionic liquid 1-allyl-3-methylimidazolium chloride for hydrophobic modification [14]; (3) CNF aerogels were coated on a silane modifier by chemical vapor deposition (CVD) [15]; (4) and CNF aerogels were modified with cationic chitosan (Ch), anionic poly(vinylphosphonic acid) (PVPA), and anionic montmorillonite clay (MMT) by a layer-by-layer technique [16]. However, these methods have complicated modification processes, and often do not pay attention to the durability of modifiers. These modifiers easily lose their functionality when they are affected by environment changes. Therefore, current research goals should focus on a simple process that is controllable and enables environmental protection. 

Water-soluble ammonium polyphosphate (APP) is an efficient commercial flame retardant with excellent performance that is non-toxic and emits no gases or drips during a flame test and has been shown to improve the flame retardancy of poly(vinyl alcohol) (PVA) aerogels [17]. However, water-soluble APP can be dissolved in water and be removed when it encounters water. This decreases the durability of APP in aerogels for practical applications. If aerogels are given hydrophobicity, this problem can be solved. Currently, some hydrophobic aerogels were proposed in a process where acid-hydrolyzed methyltrimethoxysilane (MTMS) sol modified CNF suspensions were generated via freeze-drying [18]. This modification was carried out in the aqueous phase without chemical post-treatment, which improved the hydrophobicity and mechanical properties of the CNF aerogel. MTMS has strong hydrolysis activity and good chemical stability that can be used in the reaction under aqueous conditions. It provides the conditions necessary so that the modification of MTMS and APP can be carried out simultaneously under aqueous conditions, which avoids the complicated modification process. On the other hand, the MTMS modification can effectively improve the durability of APP, providing a possibility for the application of aerogels in the field of thermal insulation.

Although numerous studies have reported on cellulose aerogels for thermal insulation materials with various water and fire resistance modifications, few have focused on using holocellulose nanofibrils (HCNF) as raw materials to prepare aerogels. Cellulose aerogels most commonly have been prepared from CNF, which uses high-pressure homogenizers. However, the energy consumption of this kind of preparation is very high and causes easy clogging of the homogenizers. Thus, the use of various pre-treatment methods, such as oxidation by 2,2,6,6-tetramethylpiperidiny loxyl or cellulose modification by the introduction of charged groups [19], were necessary for commercial exploitation of CNF production. However, this also created a complicated preparation process. Currently, it has been reported that a high content of hemicellulose might lead to an easy nanofiber fibrillation tendency [20]. Compared to TEMPO-CNF (2.5 nm), the diameter of CNF which has been prepared by holocellulose is 4.2 nm [21]. In addition, the morphology and structure of TEMPO-CNF and holocellulose-derived CNF showed no significant difference. Using holocellulose to prepare aerogels can allow one to skip the steps of TEMPO-oxidation and alkali treatment. Also, it can greatly improve the utilization of raw materials. Thus, in this study, instead of the traditional cellulose aerogel preparation methods, the IBR-derived holocellulose was directly used as the raw material to prepare aerogels via freeze-drying. To improve the properties of the holocellulose aerogel composites, the HCNF solutions were freeze-dried in the presence of APP and acid-hydrolyzed MTMS, resulting in ultralight aerogels with good hydrophobicity and fire resistance. The mechanical, thermal, hydrophobic, and flame properties of the modified aerogels were characterized, in addition to their thermal insulative properties. This eco-friendly method for the preparation of cellulose-based thermal insulation aerogels from IBR not only simplified the preparation of cellulose aerogels, but also shows its practical application prospects.

## 2. Materials and Methods

### 2.1. Materials

Industrial bamboo residues (IBR) were collected from Youzhu Technology Co. Ltd.(Yongan, China) without further treatment. Water-soluble APP (76%) was purchased from Shandong Usolf Chemical Technology Co. Ltd. (Qingdao, China). Glacial acetic acid was obtained from Beihua Fine Chemicals Co., Ltd. (Beijing, China). Sodium chlorite (80%) and MTMS (98%) was obtained from Aladdin Chemistry Co. Ltd. (Shanghai, China). Potassium bromide (KBr) and Congo red were obtained from Guangfu Technology Development Co. Ltd (Tianjin, China). All chemical reagents were used as received without further purification. Deionized water was used in all experiments.

### 2.2. Synthesis of HCNF/APP/MTMS Aerogels

The IBR was processed with chemical pretreatments of 3 wt% sodium chlorite (75 °C, 6 h) to remove the lignin, resulting in the holocellulose samples, and then, the holocellulose samples were dispersed in deionized water with a concentration of 1 wt% and nanofibrillated using industrial high-power ultrasonication (Scientz-08, Ningbo Scientz Biotechnology Co., Ltd., Ningbo, China) for 20 min at 30% power. The resulting HCNF solution was placed in a 4 °C environment before further utilization. 

Dissolving APP (0.1 g) in HCNF suspension (100 g, 1 wt%) formed APP/HCNF solutions, which were then stirred for 10 min. The obtained HCNF/APP solutions were adjusted to a pH of 4 with a 0.5 M hydrochloric acid (HCl) solution. Then, the HCNF/APP/MTMS solutions were prepared dropwise by adding MTMS (2.78 g, 20 mmol/g_(CNF)_) to HCNF/APP solutions (pH = 4) and stirred at room temperature for 2 h.

**Figure 1:**
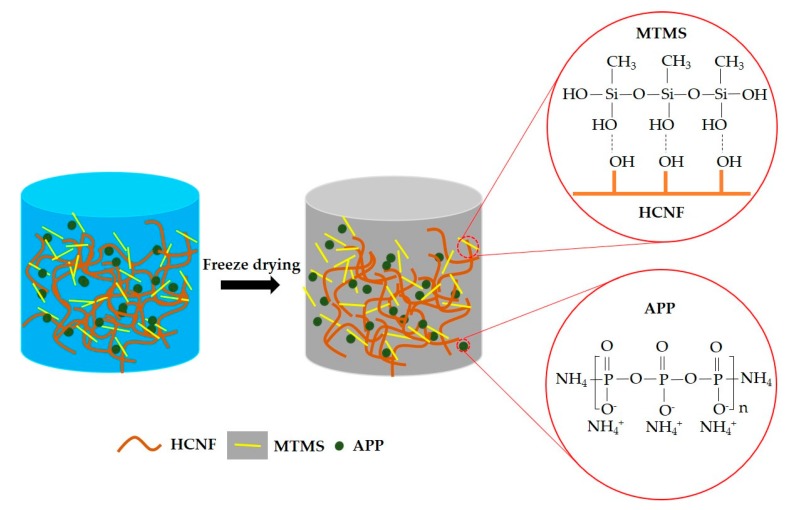
The mechanism scheme for HCNF/APP/MTMS aerogels.

As-prepared HCNF/APP/MTMS solutions were frozen in the refrigerator at −80 °C for 6 h. After freezing, the samples were immediately transferred to a freeze-drier under 0.6 Pa and −80 °C for 36 h. The obtained aerogels were sealed in plastic bags for further characterization. With respect to nomenclature, the aerogels prepared from neat HCNF solutions, HCNF/APP solutions, and HCNF/APP/MTMS solutions were called HCNF, HCNF/APP and HCNF/APP/MTMS aerogels, respectively. The mechanism scheme for HCNF/APP/MTMS aerogels is shown in Figure 1.

### 2.3. Characterization

#### 2.3.1. Chemical Composition

Before the tests, the samples were dried in an oven until the mass change was less than 0.02 g. The test methods of chemical composition on lignin, holocellulose, and α-cellulose were according to Chinese Standards of GB/T 2677-8 [22], GB/T 2677-10 [23], and GB/T 744 [24], respectively.

#### 2.3.2. Density

The densities of various aerogels were calculated by measuring their masses and dimensions. The detailed calculation uses the following equation:

(1) $$\rho =\frac{m}{V}$$

where *ρ*, *m*, and *V* are the density, quality, and bulk of aerogels, respectively.

#### 2.3.3. Scanning Electron Microscopy (SEM)

The morphologies of the aerogels were observed by scanning electron microscopy (SEM, XL30, FEI Ltd., Hillsboro, OR, USA). Aerogels were cut with a blade (Leica 819, Leica Microsystems Ltd., Wetzlar, Hessen, Germany) in liquid nitrogen, and then fixed with conductive carbon tape and coated with a platinum layer for 90 s. The morphologies were observed with an accelerating voltage of 7 kV.

#### 2.3.4. Fourier Transform Infrared (FT-IR)

Before the test, all samples were dried in an oven to eliminate the effects of moisture. The Fourier transform-infrared (FT-IR) spectra were recorded by a Nicolet IS10 FT-IR spectrometer (Thermo Fisher Scientific, Waltham, MA, USA). Aerogels were mixed in KBr with proportion of 1:100, and then ground by ball milling (ST-M200, Xuxin Instrument Co. Ltd. Beijing, China) at 1500 r/min, for 5 min. The FT-IR spectra were recorded in the range of 400–4000 cm^−1^ with a resolution of 4 cm^−1^.

#### 2.3.5. X-ray Diffraction (XRD)

A wide angle X-ray diffractometer (X PERTPRO-30X, PHILIPS Ltd., Almelo, the Netherlands) was used to determine the crystal characteristics of HCNF, HCNF/APP, and HCNF/APP/MTMS aerogels powders. The aerogel powders were smashed and sieved with more than 40 mesh. The X-rays were operated at 40 kV and 40 mA. The X-ray diffractograms were recorded at 0.02°/s over a 2θ scan in the range of 5–45°.

#### 2.3.6. Compressive Properties

The compressive properties of the aerogels (20 mm × 20 mm × 25 mm) were measured on an Instron 5848 testing machine (Instron Co. Ltd., Canton, MA, USA) with a load cell of 500 N. The stress-strain curves were measured with a compression speed of 5 mm/min to 80% strain of aerogel under a controlled atmosphere of 25 °C and 50% humidity.

#### 2.3.7. Hydrophobicity and Contact Angle

The hydrophobicity of aerogels was measured by deionized water, which was dyed with Congo red. The surface wettability of the aerogels was measured by static contact angle analysis using a contact angle goniometer (OCA20, Dataphysics Instrument, Filderstadt, Germany). The volume of the water droplet was 3 μL, and five positions were tested.

#### 2.3.8. Thermal Stability

Thermal stability measurements were obtained using a thermogravimetric analyzer (TGA, Q 50 TA Instruments, New Castle, DE, USA) from room temperature to 700 °C at a 10 °C/min heating rate under N_2_ protection. The quality of the tested aerogels was between 7–10 mg.

#### 2.3.9. Flammability and Cone Calorimetry

The flame-retardant properties were evaluated by measuring the combustion with a butane blowtorch (~1000 °C) under a fuming cupboard. The combustion of aerogels was investigated under a cone calorimeter device (FTT, Fire Testing Technology Ltd., West Sussex, RH19 2HL, UK) with heat flux of 50 kW/m^2^ in accordance with the ISO 5660-2 [25]. The aerogel (100 mm × 100 mm × 10 mm) was placed in a horizontal configuration.

#### 2.3.10. Thermal Conductivity

Thermal conductivities were tested with a hot disk thermal constant analyzer (TPS2500S, Kegonas Co. Ltd., Uppsala, Sweden), which used a transient plane source method at 24 °C. The probe (R = 3.189 mm) was sandwiched between two aerogels (100 mm × 100 mm × 10 mm) to measure the changes in temperature. The output power and time of tests were 100 mW and 10 s, respectively.

## 3. Results and Discussion

### 3.1. Characterization of HCNF, HCNF/APP, and APP/MTMS/HCNF Aerogels

**Figure 2:**
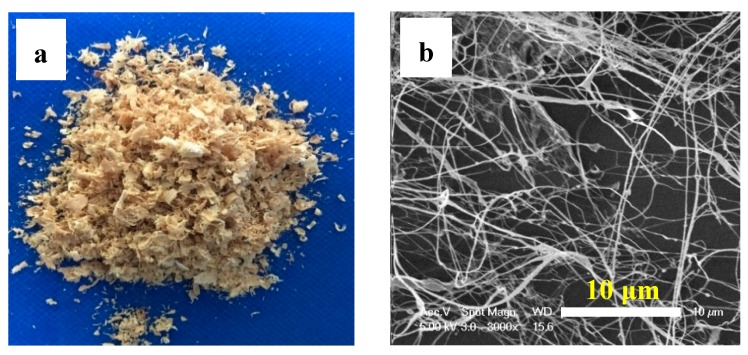
(**a**) Optical images of industrial bamboo residues (IBR) and (**b**) SEM images of as-prepared HCNF suspension with 0.01 wt%.

The morphology of the industrial bamboo residues (IBR) are shown in Figure 2a. The chemical composition of IBR is summarized in Table 1. The contents of lignin, holocellulose, and α-cellulose were 22.70, 69.08, and 39.09%, respectively. It is noteworthy that the IBR contained amounts of holocellulose, which means the utilization of IBR will be greatly improved if holocellulose can be used reasonably. Based on the results, the IBR directly went through a bleach treatment and high intensity ultrasonication without any shredding, resulting in HCNF, followed by APP and MTMS modification. The morphology of the as-obtained HCNF was characterized by SEM image (Figure 2b), indicating the HCNF was successfully prepared.

**Figure 3:**
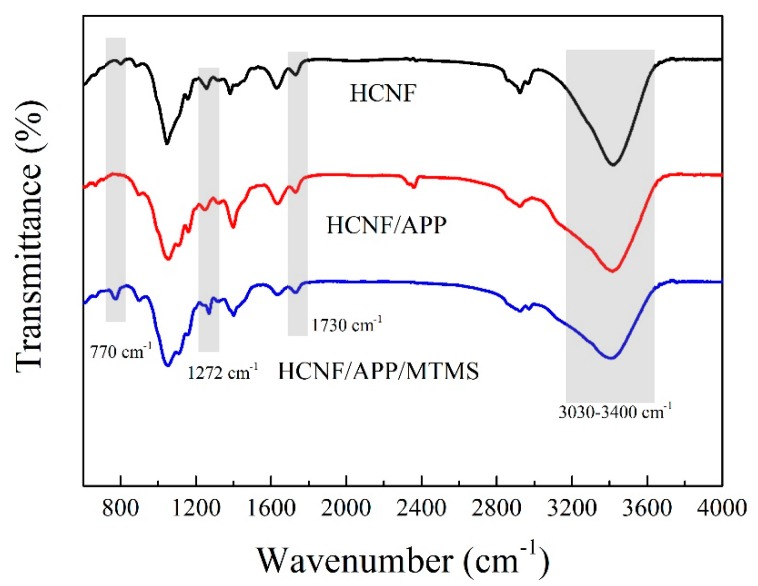
The FT-IR spectra of HCNF, HCNF/APP, and HCNF/APP/MTMS aerogels.

To investigate the modified aerogels, FTIR, XRD, and TGA were used to characterize the changes in composition and thermal stability. Figure 3 shows the FT-IR spectra of HCNF, HCNF/APP, and HCNF/APP/MTMS aerogels. The peaks at 1235 cm^−1^ and 1730 cm^−1^ in all aerogels are respectively attributed to the C=O stretching vibration in the acetyl groups and C–O stretching vibration in the glucuronic acid unit of the hemicellulose, indicating that hemicellulose exists in the aerogels [26]. In the HCNF/APP aerogels, the region at 3400~3030 cm^−1^ was broadened, which was attributed to the N–H asymmetric stretching vibration of the NH_4_^+^ in the APP [27]. This indicated that APP was homogeneously distributed in the aerogels. A new band, which indicated stretching vibrations of Si-C/Si-O-Si at ca. 770 cm^−1^, appeared in the HCNF/APP/MTMS aerogels, and the C–H deformation vibrations of –CH_3_ at ca. 1272 cm^–1^ increased significantly. Meanwhile, the amount of –OH in the HCNF/APP/MTMS aerogel was shown with a dramatic decline in the stretching vibration peak of –OH observed at ca. ~ 3314 cm^–1^ [28]. This behavior was similar to that of MTMS-modified oil-water separation materials [18]. 

**Figure 4:**
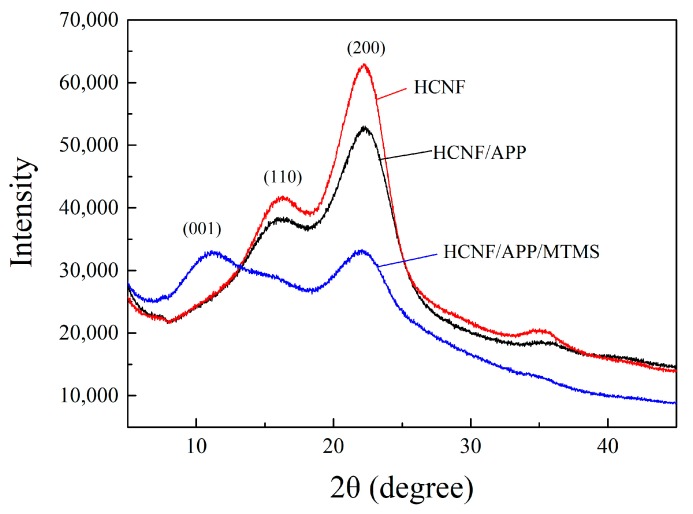
The XRD pattern of HCNF, HCNF/APP, and HCNF/APP/MTMS aerogels.

The XRD patterns of HCNF, HCNF/APP, and HCNF/APP/MTMS aerogels are shown in Figure 4. The two main diffraction peaks appeared at 2θ = 16.5°, 22.5°, and 34.6° in HCNF and HCNF/APP aerogels, which represent the crystalline area (110, 200 and 004) in the cellulose I pattern [29]. Thus, the cellulose I crystal integrity was maintained with the addition of APP. Note that with the HCNF/APP/MTMS aerogels, an additional strong diffraction peak near 2θ ≈ 10° appeared. The new peak covered the (110) crystalline area in the cellulose I crystal. However, the positions of the diffraction peaks that belonged to the cellulose I crystal were not changed. This indicates that the cellulose I crystal was also not affected by the modification of MTMS. Furthermore, the new diffraction peaks strongly resemble that of organic-inorganic phyllosilicates (001) [30], which shows the existence of MTMS.

**Figure 5:**
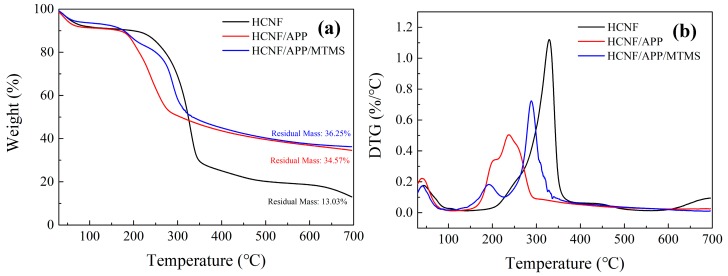
The TGA (**a**) and DTG (**b**) curves of HCNF, HCNF/APP, and HCNF/APP/MTMS aerogels.

The TGA and DTA curves of HCNF, HCNF/APP, and HCNF/APP/MTMS aerogels are shown in Figure 5, and the degradation data is summarized in Table 2. It was observed that the temperature at which 30% weight loss of the HCNF/APP aerogels occurred was decreased compared to HCNF aerogels. The lower values in both T_30%_ and T_MAX_ of HCNF/APP aerogels was main due to water-soluble APP decomposing when heated to 180 °C, leading to dehydration and carbonization of the HCNF substrate in the aerogels. However, the pyrolysis rate of the HCNF/APP aerogels was significantly decreased, and the value of residue at 700 °C increased from 13.03% to 34.57%. The heat decomposition of APP could dehydrate and carbonize the aerogel matrix to produce C=C and N−P−C structures. This could result in a charred layer, which could hinder heat transfer and prevent further decomposition [31]. It is noteworthy that, compared to HCNF/APP aerogel, the thermal stability at 30% weight loss was further improved in HCNF/APP/MTMS aerogels. Tjos could be related to the high heat stability of MTMS. From a previous report, it is known that the weight loss could eliminate the low-molecular-weight species which adsorbed at the surface of cellulosic substrate if the polysiloxane is not bonded strongly to the CNF substrate [32]. Furthermore, the effective coating of the cellulosic substrate by a modifier can form an excellent physical barrier to prevent CNF from combustion [33]. Thus, the behaviors seen in this study, in which the temperature at 30% weight loss of HCNF/APP/MTMS aerogel was significantly delayed, indicate that the improvement of thermal stability was assigned to the inherent heat resistance of the polysiloxane bonded at the HCNF surface. Moreover, the Si–O–C solid residues formed by the modified aerogel at high temperatures further enhance the effects of carbon sequestration, making the mass residues increase to 36.25%. 

**Figure 6:**
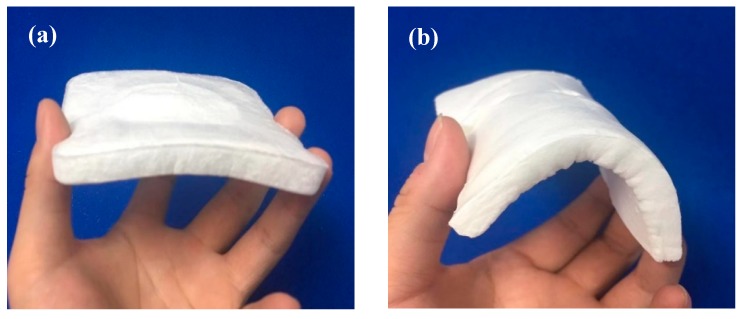
Optical images of the (**a**) HCNF aerogel, and (**b**) HCNF/APP/MTMS aerogel with different features.

**Figure 7:**
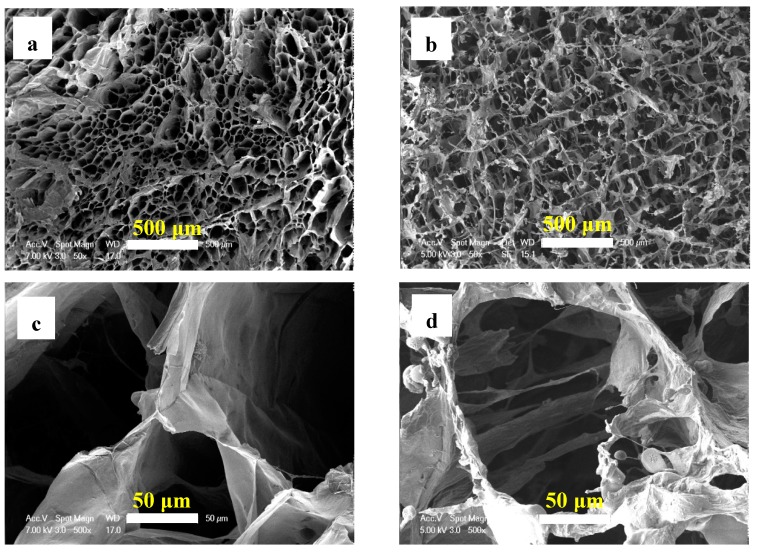
SEM images of the HCNF (**a**,**c**) and HCNF/APP/MTMS (**b**,**d**) aerogels.

To further study the properties of the modified aerogels, the morphology of HCNF and HCNF/APP/MTMS aerogels are shown in Figure 6. The HCNF/APP/MTMS aerogels were more soft and flexible than HCNF aerogels. It was indicated that the structure of HCNF/APP/MTMS aerogels clearly changes. The aerogel structures were analyzed by SEM images and are shown in Figure 7. The macropores were clearly observed in both aerogels. However, from HCNF to HCNF/APP/MTMS aerogels, the foam-like structure translates into a 3D network structure and an amount of inorganic particles coated on the surface of the aerogel matrix. Interestingly, HCNFs and bamboo fibers were intertwined, forming a porous structure in both aerogels (Figure 7c,d). This is primarily due to the structural differences of the fibers and parenchymal cells of bamboo [8]. Under the same ultrasonic treatment conditions, the parenchymal cells are more easily microfibrillated than bamboo fibers. 

### 3.2. Compressive Properties 

**Figure 8:**
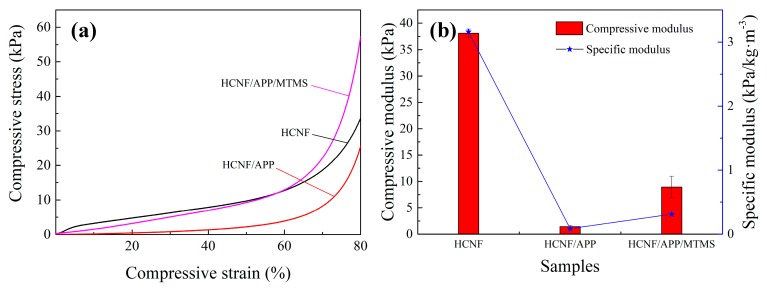
The (**a**) stress-strain curves and (**b**) compressive moduli and specific moduli of the HCNF, HCNF/APP, and HCNF/APP/MTMS aerogels.

Mechanical properties are important for thermal insulation materials. The densities of the HCNF, HCNF/APP, and HCNF/APP/MTMS aerogels were 12.04 (0.25), 16.27 (0.29), and 28.54 (0.50) kg/m^3^, respectively. The compressive properties of HCNF, HCNF/APP, and HCNF/APP/MTMS aerogels are shown in Figure 8. The typical compression curves can be divided into three stages, namely the elastic, yield, and densification stages. From the stress-strain curves, a distinct linear elastic region can be observed in the HCNF aerogels, and those of the HCNF/APP and HCNF/APP/MTMS aerogels were not boundaries between the elastic and yield regions (Figure 8a). These kinds of behaviors are similar to the compression curves of other porous materials [34]. 

The compressive modulus is the slope of the linear region of the stress-strain curve in the elastic stage. In this study, the compressive modulus can be determined at strains below 5%, because the aerogels are elastic in this stage. As shown in Figure 8b, a compressive modulus of 38 kPa was determined for the HCNF aerogels at a density of 12.04 kg/m^3^. However, the compressive modulus of the HCNF/APP aerogels was only 1.4 kPa at density a 16.27 kg/m^3^, and that of the HCNF/APP/MTMS aerogels was 8.9 kPa at a density of 28.54 kg/m^3^. There was a substantial reduction compared to the HCNF aerogels. Based on the structural changes of the aerogels, with the addition of the APP and MTMS, the foam-like structure translates into a 3D network structure, which indicated that the bonding points of substrate showed a substantial reduction in the HCNF/APP/MTMS aerogels, causing a weak crosslinking between HCNFs. The tightly-bound foam-like structure results in a relatively high resistance to compression, and the weak bonding in the 3D network structure induces lower stress values at the same deformation [35]. Thus, the mechanical properties of HCNF/APP/MTMS aerogels decreased clearly. To further understand the influence of APP and MTMS on modified fibers against density increase, the specific moduli of HCNF, HCNF/APP and HCNF/APP/MTMS aerogels were measured by using densities for normalization. The specific modulus of the HCNF aerogel was 3.16 kPa/(kg/m^3^), and that of HCNF/APP and HCNF/APP/MTMS aerogels decreased to 0.086 and 0.312 kPa/(kg/m^3^), respectively, which shows a substantial reduction. It was indicated that the APP and MTMS seriously affected the crosslinking of HCNFs, which proved the above conclusion. However, the specific moduli of HCNF/APP/MTMS aerogels were better than that of HCNF/APP aerogels, which could be due to the silane layer bonding enhancing the skeleton structure of aerogels, making HCNF/APP/MTMS aerogels have better mechanical properties than HCNF/APP aerogels.

### 3.3. Hydrolytic and Flame Resistance

**Figure 9:**
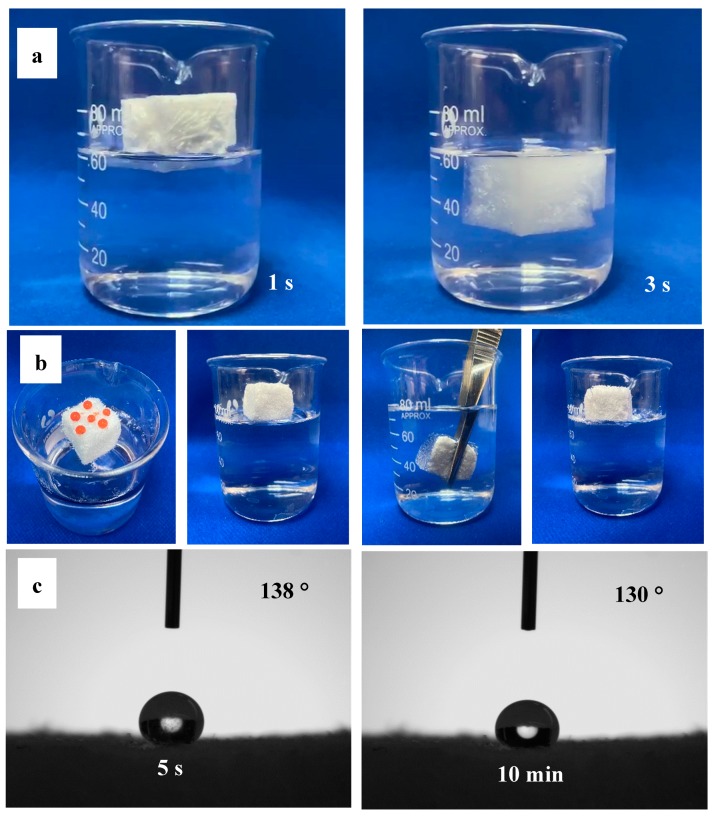
Hydrophobicity of (**a**) HCNF and (**b**) HCNF/APP/MTMS aerogels, and (**c**) water contact angles of the HCNF/APP/MTMS aeroge measured at 5 s and 10 min.

For thermal insulation materials, water and fire resistance are important. Water seepage can enable thermal insulation materials to lose insulative properties. Additionally, combustible thermally-insulative materials can easily to form a “chimney effect” so that the fire cannot be controlled. To research the application feasibility of thermal insulation materials, hydrophobic and fire resistance properties were utilized. As shown in Figure 9a, the HCNF aerogels, which contain abundant hydroxyl groups, quickly absorbed water and sunk in 3 s. However, from Figure 9b, the HCNF/APP/MTMS aerogels became hydrophobic as water formed droplets on the surface, and the aerogels could float on water. Furthermore, a HCNF/APP/MTMS aerogel could refloat on water when it was pressed into the water completely. Thus, the HCNF/APP/MTMS aerogels exhibited good hydrophobic properties. Furthermore, by the contact angle test, the 3 μL water droplet on the surface of the modified aerogel maintained a round shape with high contact angles of 130° for more than 10 min (Figure 9c). This was primarily due to the MTMS covering the HCNF surface, causing the aerogel to form an air shield at the interface between the water and the aerogel. The reduced availability of free hydroxyl groups in the matrix can effectively reduce water absorption [36]. Thus, the HCNF/APP/MTMS aerogel exhibited good water resistance, and meanwhile proved that the aerogel was well bound to MTMS. This behavior not only protects the aerogel from water damage, but also solves the problem of the dissolution of APP in water.

Moreover, the high-temperature fire resistance properties of the aerogel were assessed using a butane blowtorch (~1000 °C). It was observed that the HCNF aerogels immediately burned and the structure collapsed when contacted by fire until complete carbonization (Video S1). However, the HCNF/APP/MTMS aerogels did not burn when they came in contact with fire by butane blowtorch. Furthermore, during 60 s of fire treatment at 1000 °C, the HCNF/APP/MTMS aerogels slowly carbonized from the side near the fire source. After the flame treatment, the fire did not spread to the remainder of the aerogel (Video S2). This is due to the decomposition of APP, enabling the aerogel to form a char layer quickly. Meanwhile, the released NH_3_ and H_2_O diluted the flammable gas and oxygen to prevent further flame spreading [37]. 

**Figure 10:**
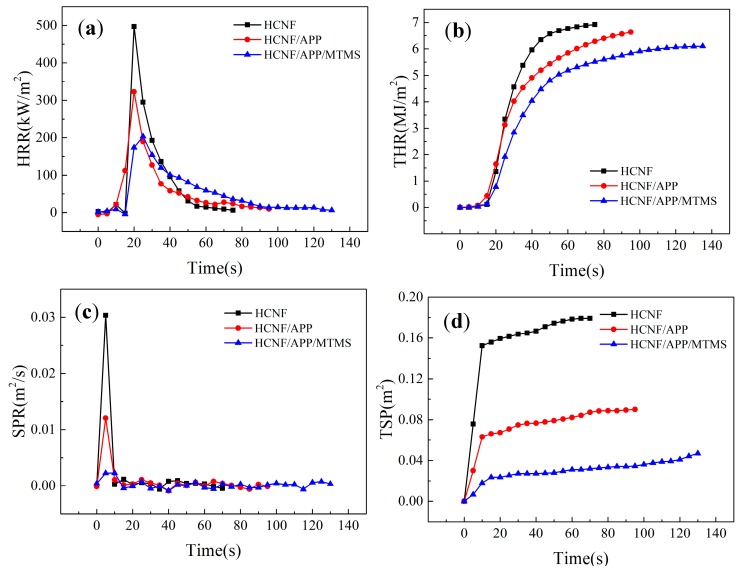
The (**a**) HRR, (**b**) THR, (**c**) SPR, and (**d**) TSP curves of the HCNF, HCNF/APP, and HCNF/APP/MTMS aerogels.

Cone calorimetry has been widely used for flame resistance testing and can provide significant amounts of data, including time to ignition (TTI), release rate (HRR), total heat release (THR), smoke production rate (SPR), and total smoke production (TSP). To gain insight into fire resistance, a cone calorimeter device, according to the standard of ISO 5660-2, was used in this study. As seen in Table 3, the TTI was increased from 0 (burns as soon as it is ignited) to 3 s after APP and MTMS modification. Although all samples were flammable under a heat flux of 50 kW/m^2^, the addition of APP and MTMS extended the TTI of the samples. As shown in Figure 10a, the HRR of HCNF/APP/MTMS aerogels was observably reduced compared with HCNF aerogels. The peak heat release rates (pHRR) of the HCNF, HCNF/APP aerogels were measured to be 466.6k W/m^2^ and 341 kW/m^2^, respectively, and that of the HCNF/APP/MTMS aerogels was 219.1 kW/m^2^, which showed a 47% decrease. Moreover, the THR of the HCNF/APP/MTMS sample was reduced from 6.9 to 6.1 MJ/m^2^ (Figure 10b). This is due to the decomposition of APP caused the high efficiency carbonization effect leading to incomplete combustion of the aerogels during testing.

**Figure 11:**
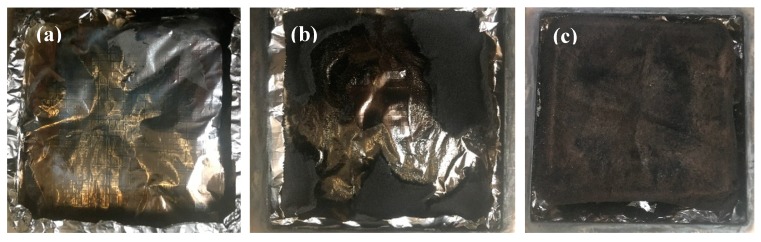
Digital photographs of residues of (**a**) HCNF, (**b**) HCNF/APP, and (**c**) HCNF/APP/MTMS aerogels after the cone calorimeter test.

SPR and TSP are important factors for insulation materials. Figure 10c,d show SPR and TSP curves during sample burning. It can be clearly observed that the SPR and TSP decreased with the addition of APP and MTMS. The peak of smoke production (pSPR) decreased from 0.024 to 0.006 m^2^/s, and TSP decreased from 0.18 to 0.04 m^2^. Together with Figure 11, it can be seen that there was no residue after cone calorimeter testing from the HCNF aerogels, while a little residue and a char layer were obtained after cone calorimeter testing from the HCNF/APP aerogels. However, more continuous char layers and the stable porous structure was preserved in the HCNF/APP/MTMS aerogels after cone calorimeter testing. These behaviors showed that the APP and MTMS played a key role in enhancing the fire resistance of HCNF/APP/MTMS aerogels. The continuous char layer can form a protective layer to prevent the spread of flame, ensuring that the cellulose skeleton is not destroyed and produces less smoke [38]. And the stable porous structure of the HCNF/APP/MTMS aerogels can effectively absorb some of the smoke [39]. 

The aerogel fire growth rate (FIGRA) is given in Table 3. FIGRA is an important parameter used to assess fire risks. The lower the FIGRA index, the higher the possibility that humans are able to survive in a fire. For the HCNF/APP/MTMS aerogel, the FIGRA index decreased from 23.3 to 8.76 kW/s·m^2^. Compared to the HCNF aerogel, the HCNF/APP/MTMS aerogel provides better chances for human survival in a fire incident.

### 3.4. Thermal Insulation Properties

Thermal conductivity is an important parameter for thermal insulation materials. The total thermal conductivities of HCNF and HCNF/APP/MTMS aerogels were measured using a hot disk thermal constant analyzer at room temperature. The results are given in Table 4.

The aerogels made from HCNF aerogels with a bulk density of 12.04 kg/m^3^ had a thermal conductivity of 0.0285 W/m·K, slightly higher than the thermal conductivity of air (0.023 W/m·K). The thermal insulative properties of HCNF aerogels are believed to be due to morphology and molecular structure. Due to the fact that HCNF aerogels exhibit low density and high porosity, the thermal conductivities of the solids in the HCNF aerogels were decreased. Furthermore, with the addition of APP and MTMS, the thermal conductivities of HCNF/APP/MTMS aerogels were increased to 0.039 W/m·K. The thermal conductivities were normalized by density to measure the specific thermal conductivity of HCNF and HCNF/APP/MTMS aerogels. The specific thermal conductivity of the HCNF aerogel was 0.0024 (W/m·K)/(kg/m^3^), and that of HCNF/APP/MTMS aerogels was 0.0014 (W/m·K)/(kg/m^3^). These behaviors indicated that the density of HCNF/APP/MTMS aerogels of up to 28.54 kg/m^3^ is the main reason for the increase of thermal conductivity. In addition, the APP and MTMS changed the structure of the HCNF/APP/MTMS aerogels, making it form an opening 3D network structure. This disordered structure with a macropore (>70 nm) can reduce the mean free paths of air molecules [40]. On the whole, the thermal conductivities of the HCNF and HCNF/APP/MTMS aerogels were in the range in 0.0285 to 0.039 W/m·K. For comparison, the expanded polystyrene of 0.030–0.045 W/m·K, and conventional biomass-derived materials such as wood or hemp fiber insulation boards, have thermal conductivities of 0.040 W/m·K or higher [41]. Moreover, conventional biomass-derived materials have poor water and fire resistance, while expanded polystyrene, although it has less water absorption, still has poor fire resistance. Thus, HCNF/APP/MTMS aerogel with ultralight, hydrophobic, and fire resistant properties makes it possible to prepare ultralight and functional thermal insulation materials in a manner which can easily be exploited in the field of building energy efficiency.

## 4. Conclusions

In this study, holocellulose aerogels were successfully prepared by freeze-drying, using IBR-derived holocellulose which allows one to skip steps of alkali treatment, and greatly improves the utilization of raw materials compared to regular CNF aerogels. Followed by APP and MTMS modification, the aerogels were given properties of hydrophobicity and fire resistance. All aerogels had low densities, ranging from 12 to 28 kg/m^3^, including the thermal conductivity of 0.028–0.039 W/m·K. The mechanical properties, microstructures, chemical compositions, hydrophobicities, thermal stabilities, and flame retardancies of the aerogels were determined. The FTIR, XRD, and TGA analyses have shown that APP and MTMS were effectively attached by the aerogels’ substrates. The HCNF/APP/MTMS aerogels showed decreased compressive strength, which was due to the structural changes after APP and MTMS additions. However, the HCNF/APP/MTMS aerogels showed higher hydrophobicity, with a contact angle up to 130° and the ability to float on water. Moreover, the fire tests, as measured by butane blowtorch (~1000 °C), showed tjat the HCNF/APP/MTMS aerogels exhibit good flame retardancy. The pHRR of 219.1 kW/m^2^, pSPR of 0.006 m^2^/s, and TSP of 0.04 m^2^ were characterized by cone calorimetry. Furthermore, the FIGRA index decreased from 23.3 to 8.76 kW/s·m^2^, which indicates that the HCNF/APP/MTMS aerogels should exhibit better performance during fire incidents. As described above, the HCNF/APP/MTMS aerogels solved problems such as poor water and fire resistance, and meanwhile solved the problem that flame retardants can be easily lost in water, showing improvement in service durability. The as-prepared aerogels could be useful for energy efficient building.

## Figures and Tables

**Table 1:** The chemical composition of industrial bamboo residues.

Sample	Lignin (%)	Holocellulose (%)	α-Cellulose (%)
Industrial bamboo residues	22.70 ± 0.66	69.08 ± 0.22	39.09 ± 0.10

**Table 2:** TGA data of HCNF, HCNF/APP, and HCNF/APP/MTMS aerogels.

Samples	T_30%_^1^ (°C)	T_MAX_^2^ (°C)	Residual Mass ^3^(%)
HCNF	298.26	329.87	13.03
HCNF/APP	236.62	237.49	34.57
HCNF/APP/MTMS	282.47	288.46	36.25

^1^ T_30%_ is temperature at 30% weight loss of aerogels. ^2^ T_MAX_ is temperature at the maximum rate of aerogel degradation. ^3^ Residual mass after heating up to 700 °C.

**Table 3:** Cone calorimeter data of the HCNF, HCNF/APP, HCNF/APP/MTMS aerogels.

Samples	TTI (s)	pHRR (kW/m^2^)	T_pHRR_ (s)	FIGRA (kW/s·m^2^)	THR (MJ/m^2^)	pSPR (m^2^/s)	TSP (m^2^)
HCNF	0 ^1^	466.6	20	23.30	6.9	0.024	0.18
HCNF/APP	1	341.0	20	17.05	6.6	0.013	0.09
HCNF/AP-P/MTMS	3	219.1	25	8.76	6.1	0.006	0.04

^1^ the HCNF aerogel was immediately burned, so the TTI cannot be measured.

**Table 4:** The thermal conductivity of HCNF, and HCNF/APP/MTMS aerogels.

Samples	Temperature (°C)	Thermal Conductivity (W/m·K)	Standard Deviation	Specific Thermal Conductivity (W/m·K)/(kg/m^3^)
HCNF	25	0.0285	0.0055	0.0024
HCNF/APP/MTMS	25	0.0398		0.0014

## Supplementary Materials

The following are available online at https://www.mdpi.com/1996-1944/13/2/477/s1, Video S1: The fire test of the HCNF aerogel by a butane blowtorch, Video S2: The fire test of the HCNF/APP/MTMS aerogel by a butane blowtorch.
